# Paeonol Ameliorates Ovalbumin-Induced Asthma through the Inhibition of TLR4/NF-*κ*B and MAPK Signaling

**DOI:** 10.1155/2018/3063145

**Published:** 2018-08-15

**Authors:** Yongjun Tang, Weihua Huang, Qianqian Song, Xiangrong Zheng, Ruohui He, Jie Liu

**Affiliations:** ^1^Department of Clinical Pharmacology, Xiangya Hospital, Central South University, Changsha, Hunan 410008, China; ^2^Institute of Clinical Pharmacology, Central South University, Hunan Key Laboratory of Pharmacogenetics, Changsha, Hunan 410078, China; ^3^Department of Pediatrics, Xiangya Hospital, Central South University, Changsha, Hunan 410008, China

## Abstract

Asthma is a chronic inflammatory disease of the airways, with complex signaling pathways involved in its pathogenesis. It was reported that paeonol attenuated airway inflammation of ovalbumin (OVA)-induced mice. Therefore, it is of importance to further investigate the underlying mechanism. BALB/c mice were challenged with OVA for the asthma model, which was validated by the changed levels of IL-4, IFN-*γ*, and IgE. The elevation of IL-4 and the decreasing of IFN-*γ* were significantly in middle (p<0.05) or high (p<0.01) paeonol dose groups compared with OVA group. MIP-1*β* in bronchoalveolar lavage fluid (BALF) also decreased significantly in middle and high paeonol group compared with OVA group (p<0.01), which is similar to the change of its mRNA in lung tissues. Moreover, the inflammatory cells infiltration and collagen deposition were attenuated by paeonol and montelukast sodium via histology examination. At last the immune blot of the protein extracted from lung tissues demonstrated that paeonol decreased the expression of TLR4 and the nuclear translocation of NF-*κ*B, as well as the phosphorylation levels of P38 and ERK in asthma model. In conclusion, paeonol ameliorated OVA-induced asthma through the TLR4/NF-*κ*B and mitogen-activated protein kinase (MAPK) signaling.

## 1. Introduction

Asthma, a chronic inflammatory disease of the airways, is caused by complex factors and affects more than 300 million individuals worldwide [1]. The most characterized features of asthma are airflow inflammation and airway remodeling. Immune changes, especially the ratio of the T lymphocyte subgroups (Th1/Th2), are of most important pathogenesis for asthma [2]. The interbalance of IFN-*γ* and IL-4 secreted by Th1 and Th2 lymphocyte subgroups, respectively, contributes largely to the progression of asthma. TGF-*β*1 that modulates the production of inflammatory factors, synthesis of collagen, fibrosis of epithelium, and hyperplasia of smooth muscle cells also plays a vital role in the airflow remodeling [3].

Current recommendations on asthma treatment have been summarized in the international recommendations of the Global Initiative for Asthma. In an early stage of asthma, a short-acting beta-agonist or a low-dose inhaled corticosteroid was recommended as-needed treatment only with as a controller. Montelukast and theophyllin were used as alternative controller, while tiotropium was chosen as add-on options. However, drug inefficacy and resistance occur frequently during asthma treatment. The treatment of Chinese medicine for asthma has a long history, and currently, it was utilized as the adjuvant treatment for asthma. Therefore, the investigation of pharmacological mechanism of Chinese medicine will bring light to asthma treatment.

Paeonol is a pharmacological active compound identified in the root bark of Chinese medicine* Paeonia moutan.* In the past decade, various studies have substantiated the pharmacological properties of paeonol, including sedation, antipyresis, analgesic, antitumor, antioxidation, anti-inflammation, and immunoregulation [4–6]. Recently, it was demonstrated that paeonol protected endotoxin-induced acute kidney injury via the inhibition of toll-like receptor 4 (TLR4) and nuclear factor kappa B (NF-*κ*B) signal pathway [7]. Further in* vitro* assay revealed that the metabolites of paeonol had stronger anti-inflammatory and antioxidant effects [8]. Although Qiang du et al. [9] have founded that paeonol possessed the therapeutic effects for allergic bronchial asthma, the specific mechanism was still largely unknown.

## 2. Materials and Methods

### 2.1. Materials

Paeonol, montelukast sodium (Mon), and ovalbumin (OVA) were purchased from Sigma-Aldrich (St. Louis, MO, USA). The primary antibody of P38, p-P38, NF-*κ*B, glyceraldehyde-3-phosphate dehydrogenase (GAPDH), and p-ERK was purchased from Cell Signaling (USA). Anti-Macrophage Inflammatory Protein 1 beta (MIP-1*β*) antibody was obtained from Abcam (USA). The primary antibody of *α*-tubulin, ERK, and cytochrome c oxidase subunit II (COX-2) was purchased from Proteintech (Wuhan, Hubei, China). All secondary antibodies were obtained from Santa Cruz. Enzyme-linked immunosorbent assay (ELISA) kits for IL-4, IFN-*γ*, MIP-1*β*, TGF-*β*, and immunoglobulin E (IgE) were from Nanjing Jiancheng Bioengineering Institute (Nanjing, Jiangsu, China).

### 2.2. Animal Experiment

**Figure 1:**
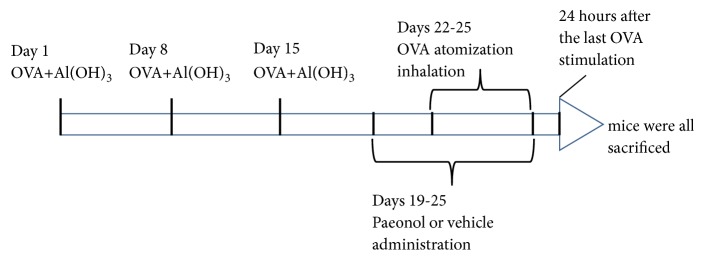
The procedures for animal experiment. The mice were challenged with intraperitoneal injection of 200 *μ*g OVA and 1 mg aluminium hydroxide on the first, eighth, and 15th day. From the 19th day, mice were continuously given intragastric administration of 0.4 ml peanut oil or drugs dissolved in peanut oil 0.5 hour for seven days. From day 22 to day 25, mice were placed into an incompletely closed container (70 cm × 50 cm × 40 cm) and subjected to continuous dose of 2.5% OVA atomization inhalation (30 min per day). All mice were sacrificed 24 hours after the last inhalation of OVA.

BALB/c mice (6-8 weeks) were from Hunan SJA Laboratory Animal Corporation and maintained in accordance with the guidelines for the care and use of laboratory animals as issued by the Center for Scientific Study with Animal models of Central South University. The mice were acclimatized under normal environmental conditions and allowed free access to standard chow and tap water for 1 week before experimentation. All animal procedures were approved by the Ethics Committee of Central South University on Laboratory Animal Care and briefly shown in Figure 1. The mice were challenged with intraperitoneal injection of 200 *μ*g OVA and 1 mg aluminium hydroxide dissolved in 0.2 ml normal saline on the first, eighth, and 15th day. From day 22 to day 25, mice were placed into an incompletely closed container (70 cm × 50 cm × 40 cm) and subjected to continuous dose of 2.5% OVA atomization inhalation (30 min per day). From Day 19 to Day 25, mice were continuously given intragastric administration of 0.4 ml peanut oil or drugs dissolved in peanut oil 0.5 hours before OVA stimulation once a day. For experimental grouping, 48 mice were divided into 6 groups (8 per group): vehicle group (Con), OVA-administrated model group (OVA), montelukast sodium-treated group (OVA+Mon), low paeonol dose-treated group (OVA+L-paeonol; paeonol 100 mg/kg), middle paeonol dose-treated group (OVA+M-paeonol; paeonol 200 mg/kg), and high paeonol dose-treated group (OVA+H-paeonol; paeonol 400 mg/kg).

### 2.3. The Assay of Inflammatory Factors

24 hours after the last dose of stimulation, mice were anesthetized by 10% chloral hydrate and fixed. After the routine disinfection, incise the skin of mice neck, detach the layer of muscle, and expose the trachea. Then the main trachea was incised and 1 mL PBS was injected to wash the lung for three times via syringe. Recover the resulting wash liquid, record the recovery volume, and count the cell number of bronchoalveolar lavage fluid (BALF). Blood samples were also collected from mice. Serum was obtained from blood by centrifugation (3000 rpm for 10 min at 4°C). The levels of IL-4, IFN-*γ*, MIP-1*β*, and TGF-*β* in BALF and IgE in serum were measured using commercial enzyme kits according to the instructions provided by the manufacturer. The real-time qPCR was performed in duplicate using specific primers of MIP-1*β* (Forward primer: TTCCTGCTGTTTCTCTTACACCT; Reverse primer: CTGTCTGCCTCTTTTGGTCAG) and SYBR green I mixture (Takara). Relative changes in gene expression were calculated as fold changes using the comparative ΔΔCt method (where Ct is threshold cycle) using mice GAPDH housekeeper as the housekeeping gene for normalizing.

### 2.4. Immune Blot and q-PCR

Lung tissues from mice were firstly ground into powder using nitrogen and then dissolved in lysis buffer (50 mM Tris, pH 7.5, 150 mM sodium chloride, 1 mM phenyl-methylsulfonyl fluoride, 1 mM sodium orthovanadate, 1% Nonidet P-40, 50 mM sodium fluoride, 10 mg/mL proteinase inhibitors mixture, and 10% glycerol) at 4°C, followed by the centrifugation at 14,000 rcf at 4°C for 10 min. Protein concentration was measured by the Bradford assay, using bovine serum albumin as standard. Proteins (25 *μ*g/lane) were separated by SDS-PAGE and then transferred to polyvinylidene difluoride membranes (Millipore, Billerica, MA). Nonspecific binding was blocked by preincubation of the polyvinylidene fluoride (PVDF) membrane in Tris-buffered saline containing 0.1% Tween 20 (TBS-T) and 5% skimmed milk for 1 h. The PVDF was then incubated overnight at 4°C with primary antibodies. After being washed, blots were then incubated with horseradish peroxidase (HRP) labeled secondary antibody and washed before bands were revealed using the BioRad imaging system. Band densities were determined using Image Pro Plus Software. The density ratios of target proteins and GAPDH in each group were compared. Total RNA was isolated from the lung tissues using TRIzol and phenol-chloroform method. 500 ng of the total RNA was reversely transcribed using a high capability reverse transcription kit with DNA erase (Takara) and then appropriately diluted.

### 2.5. Histological Examination

The lung tissue was immersed in 4% phosphate-buffered paraformaldehyde for 24 h. Then, the lung tissues were embedded in paraffin and sliced into sections 5 *μ*m thick, which were then stained with hematoxylin and eosin (H&E) or Masson. The degree of lung inflammation was scored by two practiced pathologists from Xiangya Hospital, according to the previous study [9, 10]. The scoring system of 0-4 based on the degree of total cell infiltration was used (0: none cells infiltration, 1: few cells, 2: mild influx of cells, 3: moderate influx of cells, and 4: extensive influx of cells), with three fields for each slide.

### 2.6. Statistical Analysis

Statistical analysis was conducted by Statistical Products Social Science (SPSS) 19 for Windows. Experimental values are expressed as mean ± SEM. Comparison of mean values between groups was performed by unpaired t-test or one-way analysis of variance (one-way ANOVA) followed by post hoc Tukey test. P<0.05, P<0.01, and P<0.001 were considered to be significant.

## 3. Results

### 3.1. The Change of Inflammatory Factors and Inflammatory Cells Infiltration

The concentrations of IL-4, IFN-*γ*, TGF-*β*, and MIP-1*β* in BALF and IgE in serum were determined by ELISA, shown in Table 1. OVA stimulation significantly increased the levels of IL-4 (P<0.01), TGF-*β* (P<0.05), and MIP-1*β* (P<0.01) but decreased IFN-*γ* (P<0.01). Paeonol administration decreased IL-4 but increased IFN-*γ* levels, especially with middle and high dosage (Table 1). The increasing levels of TGF-*β* by OVA stimulation were also decreased but not significantly. It was shown in Table 1 that MIP-1*β* could be induced by OVA. Moreover, the concentrations of MIP-1*β* in BALF were significantly lower in H-paeonol (P<0.01) and M-paeonol (P<0.01) groups than in OVA. The cells infiltrated in BALF mainly contained lymphocytes, neutrophils, macrophages, and eosinophils, indicating the trend of inflammation. The number of total infiltrated cells was counted (Table 1). It was shown that OVA increased cell infiltration, whereas middle and high dose of paeonol inhibited the cell infiltration.

### 3.2. Paeonol Inhibits TLR4/ NF-*κ*B/MIP-1*β* Signaling in Mice Asthma Model

**Figure 2:**
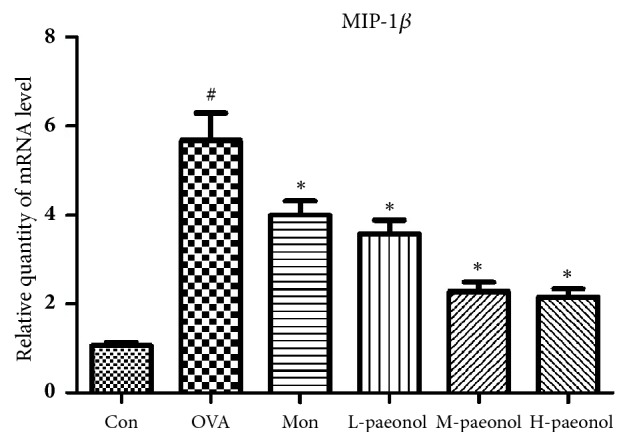
The mRNA expression of MIP-1*β* in lung tissues. The data were expressed as the means ± SEM (n = 6 per group). Macrophage Inflammatory Protein 1 beta, MIP-1*β*. #P < 0.05 compared with Con, *∗*P < 0.05 compared with OVA.

**Figure 3:**
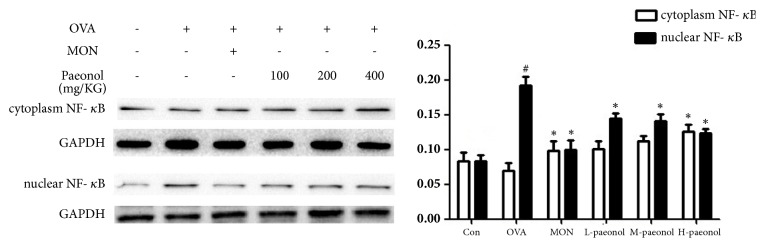
The cytoplasm and nuclear expression of NF-*κ*B in response to paeonol treatment. The data were expressed as the means ± SEM (n = 4 per group). Nuclear factor kappa B, NF-*κ*B; ovalbumin, OVA; control, Con; montelukast sodium, Mon. #P < 0.05 compared with Con, *∗*P < 0.05 compared with OVA.

**Figure 4:**
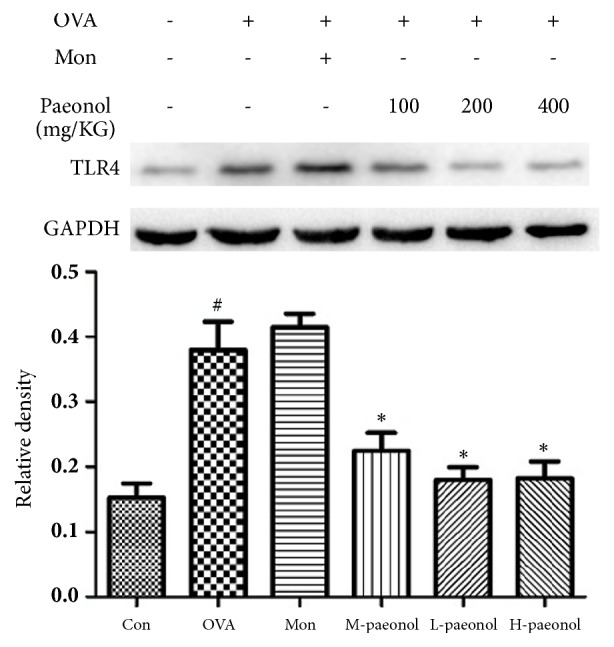
The changed expression of TLR4 subjected to paeonol treatment. The data were expressed as the means ± SEM (n = 4 per group). Toll-like receptor 4, TLR4; ovalbumin, OVA; control, Con; montelukast sodium, Mon. #P < 0.05 compared with Con, *∗*P < 0.05 compared with OVA.

MIP-1*β* is a secretory factor related to airway inflammation and the secretion of MIP-1*β* was decreased by paeonol treatment.  Figure 2 showed that the expression levels of MIP-1*β* mRNA in lung were also more high in OVA group than CON (P<0.01), and paeonol treatment significantly decreased the levels of MIP-1*β* mRNA compared with OVA (P<0.01). However, the protein band of MIP-1*β* was not detected using the total protein extracted from lung tissues. Since MIP-1*β* is a downstream signal of NF-*κ*B, we detected the nuclear translocation of NF-*κ*B using p65 antibody with the result shown in Figure 3. The nuclear translocation of NF-*κ*B was increased by OVA treatment, whereas Mon and paeonol inhibited its nuclear translocation. The protein levels of TLR4 were further detected and shown in Figure 4. OVA stimulation increased the expression of TLR4, whereas paeonol significantly decreased the TLR4 levels (P<0.01).

### 3.3. The Function of Paeonol on MAPK Pathway in Asthma

**Figure 5:**
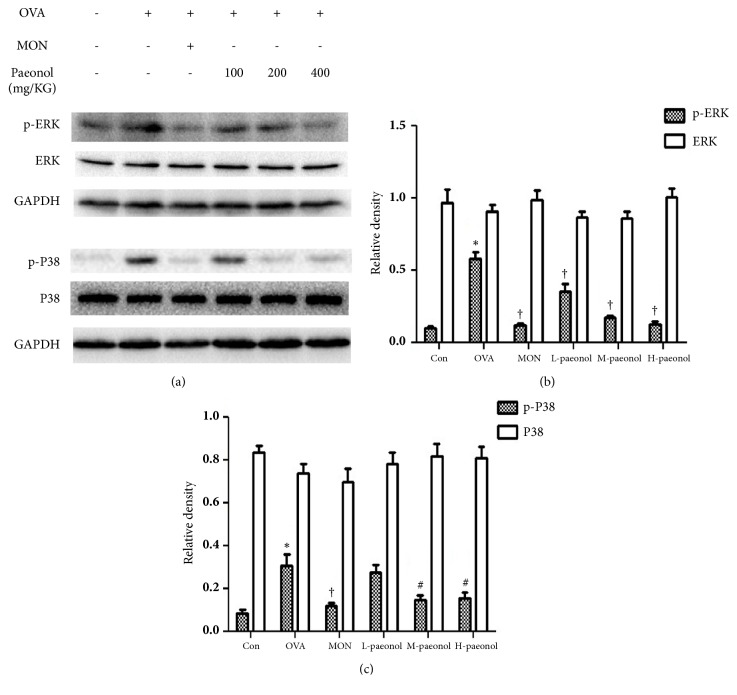
Changed expression of p-P38 and p-ERK in asthma and paeonol treatment. (a) The expression of p-ERK, ERK, p-P38, and P38 was analysed by immune blot. GAPDH was utilized as the standard control. (b) The band signal strengths of p-ERK and ERK were expressed as a ratio to ERK and GAPDH, respectively (n= 4 per group). (c) The band signal strengths of p-P38 and P38 were expressed as a ratio to P38 and GAPDH, respectively. The data were expressed as the means ± SEM (n = 4 per group). Extracellular signal-regulated kinase, ERK; glyceraldehyde-3-phosphate dehydrogenase, GAPDH; ovalbumin, OVA; control, Con; montelukast sodium, Mon. *∗*P < 0.01 compared with Con, #P < 0.05 compared with OVA, and †P < 0.01 compared with OVA.

The effect of paeonol on MAPKs was investigated to reveal its pharmacological mechanism in asthma treatment. It was shown that both the p38 and ERK protein levels did not change in any group (Figure 5). However, OVA stimulation induced the phosphorylation of both p38 and ERK, which could be reduced by paeonol, especially the high dose (Figure 5).

### 3.4. Effect of Paeonol on Airway Inflammation and Collagen Deposition

**Figure 6:**
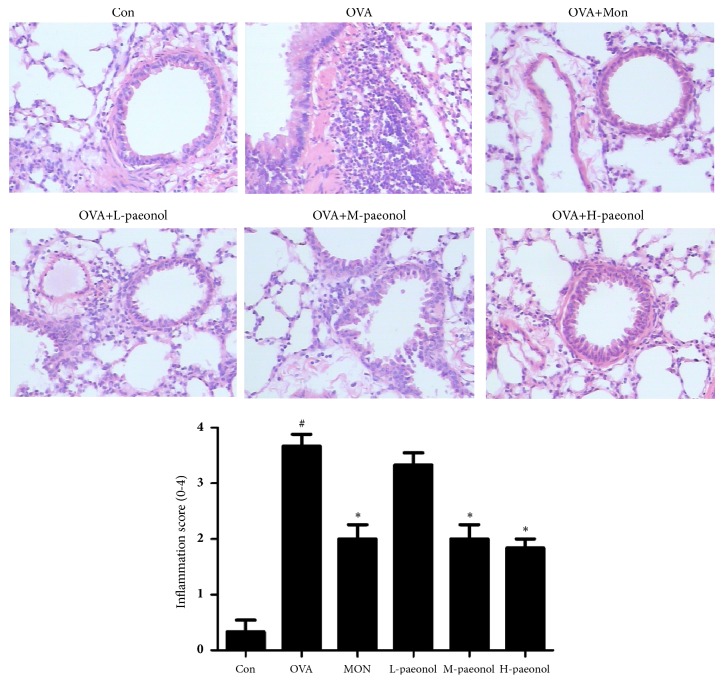
H&E staining of lung tissues and inflammation assessment. The data were expressed as the means ± SEM (n = 4 per group). ×200 magnification, #P < 0.05 compared with Con, *∗*P < 0.05 compared with OVA.

**Figure 7:**
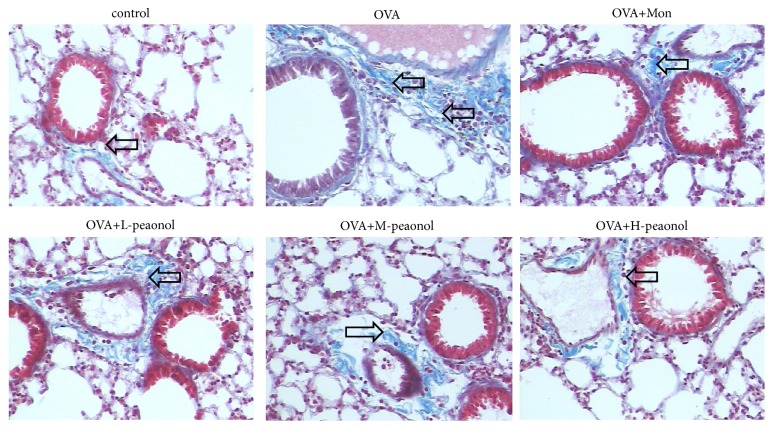
Masson staining of lung tissues used to demonstrate the extent of collagen deposition or fibrosis. Collagen fibers in lung tissue were dyed to blue and indicated by arrows, ×200 magnification.

The H&E staining of lung tissues (Figure 6) showed that inflammatory cells infiltration was largely induced in OVA group. The infiltration was decreased by Mon or paeonol (middle and high dose) administration. In lung tissues, the extent of collagen deposition/fibrosis around the airways and vessels was profoundly triggered by OVA stimulation, whereas Mon or paeonol treatment largely reduced this collagen deposition (Figure 7).

## 4. Discussion

Recently, monomers of Chinese traditional herbs have attracted more and more attention because of their desirable pharmacological effects. Many monomers from Chinese medicine were valuable in the treatment of asthma, such as baicalin [11], salidroside [12], astragalus membranaceus [13], and luteolin [14]. Although most of these monomers were investigated in murine models, their therapeutic effects shown on asthma are promising. Therefore, further studies on these monomers could facilitate the design of novel medicine for asthma therapy.* Paeonia moutan* or* Moutan Cortex* root is an old Chinese traditional medicine with a wide usage. Formulae (*e.g., peony* and* licorice* decoction) that contains* Paeonia moutan* were used to treat asthma in Chinese medicine. Paeonol, as one of the active compounds in* Paeonia moutan*, merits substantial pharmacological properties including sedation, antipyresis, analgesic, antitumor, antioxidation, anti-inflammation, and immunoregulation [8]. In the previous and present study, paeonol was shown to have therapeutic effect on airflow inflammation and airway remodeling caused by OVA.

Qiang Du et al. [9] firstly demonstrated that paeonol administration increased the level of Th1 cytokine IFN-*γ* and decreased Th2 cytokines IL-4 and IL-13. The study also revealed the changes of TGF-*β*. Indeed, it is widely accepted that the immune imbalance of Th1/Th2 plays an important role in the asthmatic morbidity mechanism. We also found the change of Th1 and Th2 cytokines (*e.g.,* IFN-*γ* and IL-4) in OVA-stimulated BALB/c mice, with the similar trends. In the regulation of cytokines, NF-*κ*B plays an important role. NF-*κ*B is activated by the proinflammatory stimuli and then translocated into the nucleus. Thereafter, it enhances the downstream signal MMP-9, increasing the inflammatory cell recruitment and releasing proinflammatory cytokines (such as IL-4, IL-5, and IL-13) [15]. TGF-*β*, which can stimulate the fibrosis and inflammation, was upregulated by OVA. TGF-*β* combined to and activated TGF-*β*1 receptors which further increased phospho-Smad levels [16]. MAPK pathways (ERK, P38, and JNK) can be activated by TGF-*β* through Smad [17, 18]. In reverse, p38 MAPK modulates T cell immunity and regulates the release of IL-4 and IgE [19, 20]. In this study, the use of paeonol largely recovered the alteration of these cytokines. As shown in the results of histopathology that the inflammatory cells infiltration and collagen deposition were significantly reduced in paeonol-treated group, paeonol can reverse OVA-induced inflammation and fibrosis.

MIP-1*β*, also known as CCL4, is mainly produced by macrophages and can combine to CCR5 receptors [21]. It is a chemoattractant for natural killer cells, monocytes, and lymphocyte [22]. Early study demonstrated that MIP-1*β* is involved in the pathology of rheumatoid arthritis [23]. In the present study, both the mRNA levels and alveolar fluid concentrations of MIP-1*β* were increased by OVA stimulation. Thus, MIP-1*β* is thought to induce inflammatory responses related to asthma. NF-*κ*B is an essential hinge that triggers the inflammation response. A number of studies have highlighted the role of NF-*κ*B in asthma. The immune blot of nuclear and cytoplasm protein revealed that paeonol suppressed the nuclear translocation of NF-*κ*B. Interestingly, the activation of NF-*κ*B has been shown to induce the production of MIP-1*β* and other inflammatory factors [23, 24]. Moreover, it was reported that paeonol suppressed TLR4 expression and NF-*κ*B signaling in kidney [7] and neurocyte [25]. The phenomenon that paeonol suppressed TLR4 expression was also observed in OVA-induced asthma model of our study. Therefore, it was possible that paeonol attenuated OVA-induced asthma by restraining TLR4/NF-*κ*B/MIP-1*β* pathway.

MAPKs pathway plays a pivotal role in the pathology and progression of asthma. With regard to LPS-induced inflammation in RAW 264.7 cells, paeonol was found to reduce the phosphorylation levels of P38 and ERK1/2 in a dose-dependent manner, but not JNK. In our study of asthma, we also found that the phosphorylation levels of P38 and ERK were downregulated by paeonol, whereas P38 and ERK were unchanged. By using airway biopsy samples from allergic asthmatic patients and healthy controls, it was also found that the phosphorylation of MAPKs was highly expressed [26]. The inhibition of MAPKs activity via pharmacological or genetic approaches blocks allergic inflammation of airways [27]. Nevertheless, the literature upon the role of MAPKs in asthmatic airways is limited. Based on the known data, we arbitrarily suggested two aspects of MAPKs in asthma pathology. For one aspect, MAPK pathways are essential for activation of various immune cells and inflammatory cytokines production under cellular stresses. It has been found that ERK1/2 can regulate IL-8 production and TNF*α* in macrophages [26, 28]. For another aspect, the cytokines lead to proliferation, differentiation, and fibrosis probably through the MAPKs signaling [29]. It is regrettable that the network of MAPKs was not fully explored in pathology of asthma.

However, the* in vivo* pathological processes of asthma are even more complicated. Bronchial asthma is chronic disorder characterized by airway inflammation, reversible airway obstruction, mucus hypersecretion, and airway hyperresponsiveness [30]. In the processes of airway inflammation during asthma, a variety of cells such as eosinophils, T lymphocytes, mast cells, neutrophils, airway smooth muscle cells, and dendritic cells are involved [31, 32]. Further study was needed to elucidate the pharmacological function of paeonol to these cells.

In summary, as an active compound identified in* Paeonia moutan*, paeonol can attenuate OVA-induced asthma in murine model. The therapeutic effect of paeonol was related to its inhibitory function on TLR4/NF-*κ*B and MAPK signaling.

## Figures and Tables

**Table 1:** The change of inflammatory factors and total inflammatory cells.

Group	IL-4 (pg/mL) in BALF	IFN-*γ* (pg/mL) in BALF	TGF-*β* (pg/mL) in BALF	MIP-1*β* (pg/mL) in BALF	Serum IgE (ng/mL)	No. of infiltrated cells in BALF (10^5^/mL)
Con	43.1±5.1	41.2±3.1	22.4±1.5	3.39±1.30	41.2±2.6	2.73±0.20
OVA	88.0±7.3#	20.5±1.9#	32.8±4.1*∗*	8.26±2.10#	88.8±5.4#	7.04±1.00#
OVA+Mon	52.0±5.7‡	30.9±3.71†	22.9±2.8	6.89±1.64	54.8±4.9‡	4.21±0.44†
OVA+L-paeonol	75.2±8.1	17.5±2.7	28.0±3.1	6.69±1.46	79.0±5.0	5.07±1.15
OVA+M-paeonol	62.8±4.6†	31.1±4.1†	24.9±4.46	5.11±1.37‡	59.2±5.2‡	2.61±0.30†
OVA+H-paeonol	53.6±6.3‡	34.8±4.3‡	24.2±5.2	4.28±1.33‡	53.7±4.9‡	3.94±1.08‡

Bronchoalveolar lavage fluid, BALF; Macrophage Inflammatory Protein 1 beta, MIP-1*β*; immunoglobulin E, IgE; ovalbumin, OVA; control, Con; montelukast sodium, Mon. n=6-8; *∗P*<0.05, #*P*<0.01, compared with Con group; †*P*<0.05, ‡*P*<0.01, compared with OVA group.
